# Simultaneous Robot‐Assisted Distal Gastrectomy and Radical Cystectomy With a Shared Port Configuration in Situs Inversus Totalis

**DOI:** 10.1002/iju5.70281

**Published:** 2026-10-14

**Authors:** Takaharu Hamada, Shugo Yajima, Shota Umezawa, Gaku Okumura, Tomoki Kimura, Shu Gozu, Madoka Kataoka, Yasukazu Nakanishi, Takahiro Kinoshita, Hitoshi Masuda

**Affiliations:** ^1^ Department of Urology National Cancer Center Hospital East Kashiwa Chiba Japan; ^2^ Department of Gastric Surgery National Cancer Center Hospital East Kashiwa Chiba Japan

**Keywords:** cystectomy, gastrectomy, multiple primary neoplasms, simultaneous robotic surgery, situs inversus

## Abstract

**Introduction:**

Situs inversus totalis complicates minimally invasive surgery, and simultaneous robotic surgery for tumors of different organs is particularly challenging.

**Case Presentation:**

A 72‐year‐old man with situs inversus totalis receiving androgen blockade for prostate cancer developed a 40‐mm muscle‐invasive bladder tumor undetected on magnetic resonance imaging 7 months earlier; synchronous gastric cancer was also diagnosed. After four chemotherapy cycles, robot‐assisted distal gastrectomy and radical cystectomy with an ileal conduit were performed in one session, sharing four of eight ports and lasting 10 h 27 min. The bladder showed no residual carcinoma (ypT0N0) and the gastric cancer ypT2N3aM0; he died 27 months postoperatively.

**Conclusion:**

Two major robotic resections were completed in one session, to our knowledge for the first time. Port sharing avoided a second set of incisions and a second port map, although the robot was undocked and redocked. The surgery was prolonged and followed by suspected non‐occlusive mesenteric ischemia.

**Keynote Message**

Eight abdominal port sites were used for two major robot‐assisted resections in a patient with situs inversus totalis; four sites, including the camera port, were shared so that a second set of incisions and a second port map were avoided; the robot was redocked onto the existing ports. The gastric ports were not mirrored, and only the table tilt was reversed. The session lasted for more than 10 h and was followed by suspected non‐occlusive mesenteric ischemia.

## 1 Introduction

Situs inversus totalis is a congenital mirror‐image transposition of the thoracic and abdominal viscera occurring in approximately one in 10 000 individuals [1]. The reversed anatomy complicates minimally invasive surgery, and the robotic platform is increasingly preferred in this setting [2]. Robot‐assisted radical cystectomy has been reported only exceptionally for this anomaly [3], and simultaneous robotic resections of two organs only once [4]. We describe robot‐assisted distal gastrectomy and radical cystectomy with an ileal conduit performed in one session, through a partly shared port configuration, in a man with situs inversus totalis and synchronous prostate, bladder, and gastric cancers.

## 2 Case Presentation

A 72‐year‐old man with known situs inversus totalis presented with urinary urgency and a prostate‐specific antigen concentration of 23.3 ng/mL. Magnetic resonance imaging showed a right peripheral zone lesion and no bladder abnormality (Figures 1 and 2a), and biopsy revealed adenocarcinoma (Gleason score 4 + 3, cT2cN0M0). Androgen blockade was initiated. Seven months after that imaging, the antigen concentration had fallen to 0.01 ng/mL, but repeat magnetic resonance imaging showed a 40‐mm muscle‐invasive tumor on the posterior bladder wall (Figure 2b). Computed tomography also showed enlarged perigastric nodes with complete visceral transposition (Figure 2c). Transurethral resection yielded a malignant small round cell tumor consistent with a carcinoma. CD56 was diffusely positive, whereas chromogranin A, synaptophysin and urothelial, prostatic, lymphoid and melanocytic markers were negative (Table 1); the tumor was regarded as a poorly differentiated carcinoma with neuroendocrine differentiation falling short of the criteria for neuroendocrine carcinoma. Endoscopy revealed a poorly differentiated adenocarcinoma of the gastric antrum. The gastric cancer determined the prognosis, and four cycles of fluorouracil, leucovorin, oxaliplatin, and docetaxel were administered [5]. Both tumors regressed (Figure 2d).

**FIGURE 1:**
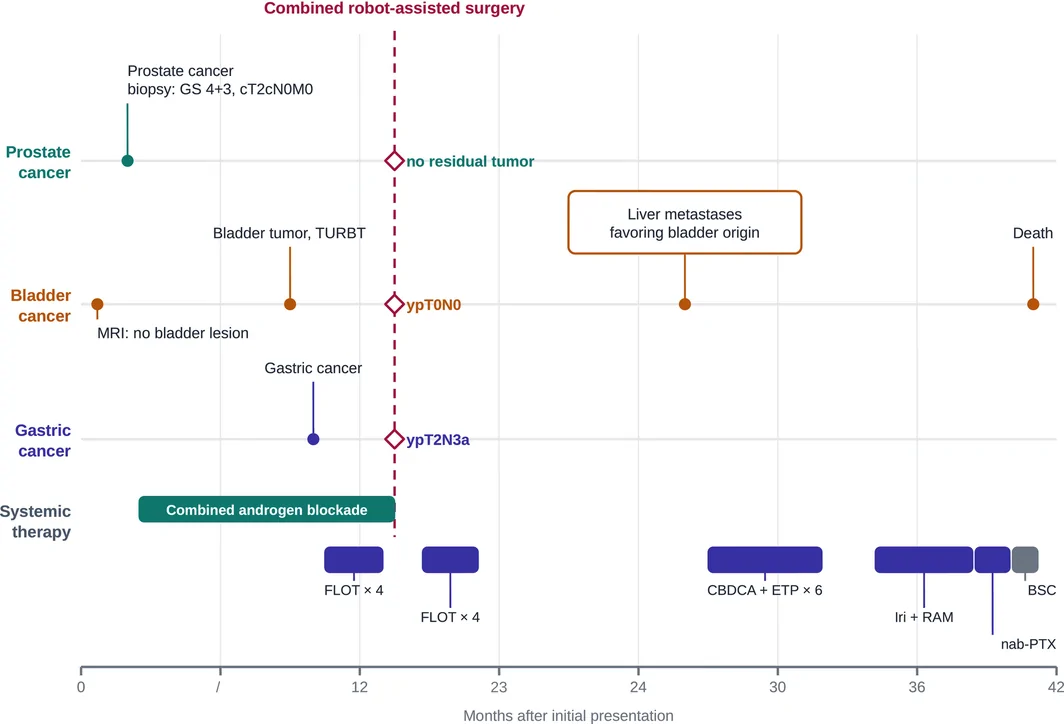
Clinical course, shown as months after the initial presentation. The broken vertical line marks the combined robot‐assisted surgery, and the open diamonds indicate the organ resected at that surgery in each disease. The label below the bladder lane marks the pelvic magnetic resonance image that showed no bladder lesion. Systemic treatments are shown as horizontal bars. BSC, best supportive care; CBDCA, carboplatin; ETP, etoposide; FLOT, fluorouracil, leucovorin, oxaliplatin and docetaxel; Iri, irinotecan; nab‐PTX, nab‐paclitaxel; RAM, ramucirumab; TURBT, transurethral resection of bladder tumor.

**FIGURE 2:**
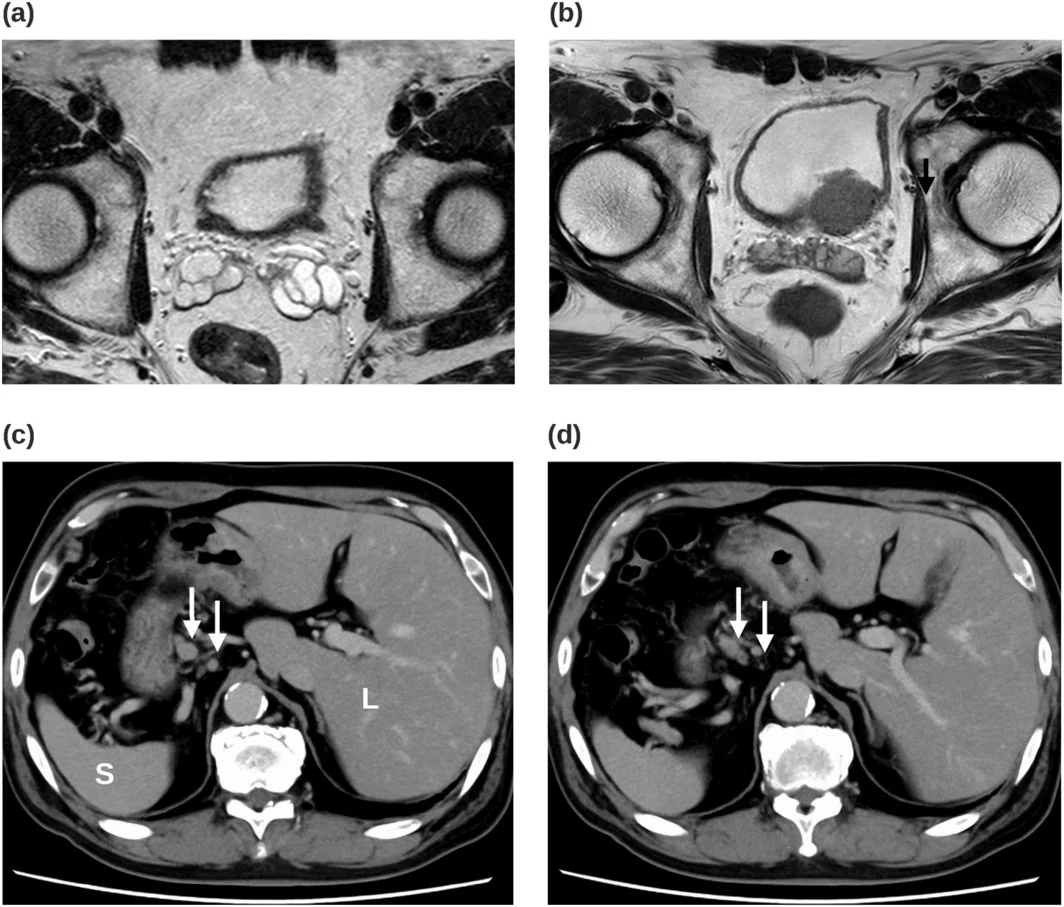
(a) T2‐weighted magnetic resonance image obtained at the time of the prostate biopsy: The bladder wall is smooth and no tumor is present. (b) The same sequence and level at 7 months later: A 40‐mm broad‐based tumor arising from the posterior wall of the bladder (arrow). (c) Contrast‐enhanced computed tomography of the upper abdomen before chemotherapy: Enlarged perigastric lymph nodes (arrows). The viscera are completely transposed; the liver (L) lies on the left and the spleen (S) lies on the right. (d) The same level after four cycles of fluorouracil, leucovorin, oxaliplatin and docetaxel: The nodes (arrows) have markedly regressed.

**TABLE 1:** Immunohistochemical profile of the bladder tumor, the gastric cancer, and the liver metastasis.

	Bladder tumor (transurethral resection)	Gastric cancer (biopsy/resection)	Liver metastasis (needle biopsy)
CK7	ND	ND	+
CK20	ND	ND	−
CAM5.2	+ (focal)	ND	+
CK AE1/AE3	+ (partial)	ND	ND
CD56	+ (diffuse)	−	+
Chromogranin A	−	ND	−
Synaptophysin	−	ND	−
INSM1	ND	ND	+
GATA3	−	−	ND
NKX3.1	−	ND	ND
HNF4α	ND	+	−
Claudin‐18	ND	+	−
TROP2	ND	+	−
p63 / p40	−/ND	ND/−	ND
Ki‐67 (MIB‐1)	> 80%	ND	approximately 70%

*Note:* CD56 was stained retrospectively on the gastric resection specimen and was negative; INSM1 was not available for the bladder specimen. The liver metastasis shared CD56 positivity with the bladder tumor and lacked the HNF4α, claudin‐18 and TROP2 expression observed in the gastric cancer, supporting a bladder rather than a gastric origin.

Abbreviation: ND, not done.

Performance status was 0; the comorbidities were hypertension, dyslipidemia and a deep vein thrombosis on apixaban. As both tumors had responded, a single session was preferred to staged resections, which would have delayed adjuvant chemotherapy and required a second anesthetic and anticoagulation interruption. Preoperative computed tomography confirmed a pure mirror image of the visceral vasculature, without variation of the hepatic arteries. Both procedures used the da Vinci Xi system (Intuitive Surgical, Sunnyvale, CA, USA) and, in total, eight port sites (Figure 3a–c), with the pneumoperitoneum maintained at 10 mmHg.

**FIGURE 3:**
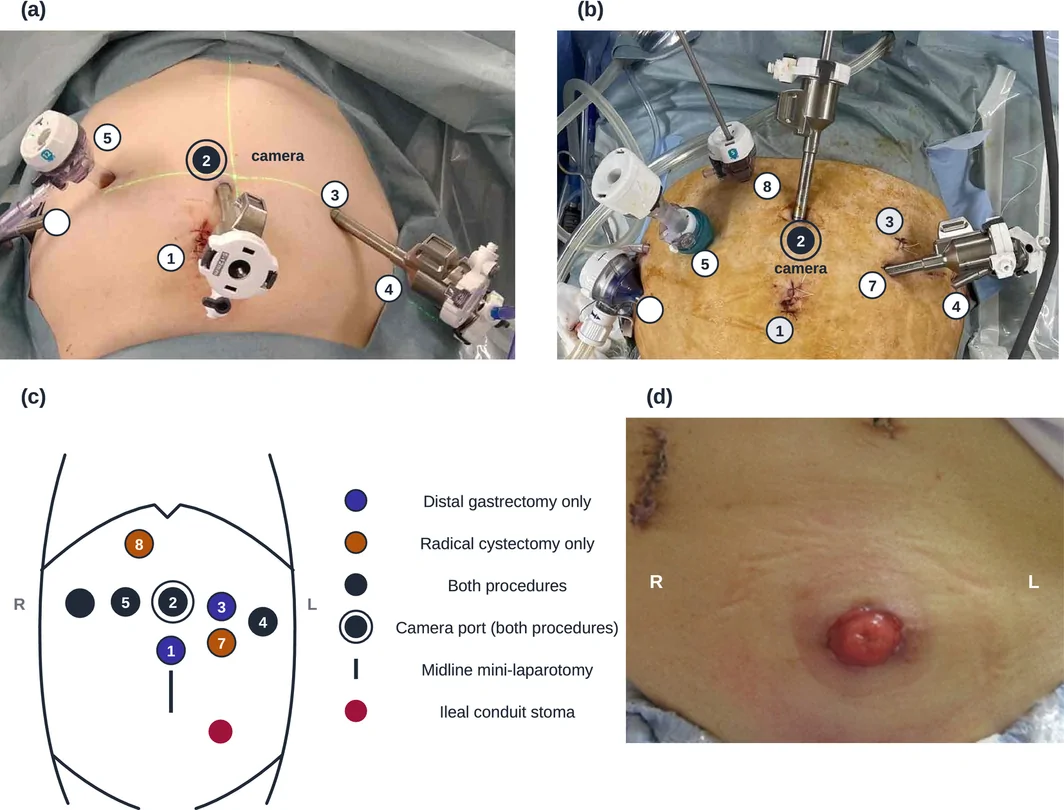
Port configuration for the two robot‐assisted procedures. (a) Port placement for distal gastrectomy and (b) radical cystectomy, photographed from the foot of the bed, so that the head of the bed is at the top and the patient's right is on the left of each image. In (b), the sites used only for the gastrectomy (1 and 3) have been closed and are shaded, and Sites 7 and 8 have been added; Site 2 served as the camera port for both procedures and is ringed. (c) Schematic of the eight port sites, with the abdominal wall seen from the front; R and L denote the patient's right and left. Site 1 is the umbilicus, opened to establish pneumoperitoneum. For the gastrectomy, Site 2 was an 8‐mm camera port, sites 3, 4 and 6 were 8‐mm instrument ports, and Site 5 a 12‐mm assistant port. For the cystectomy, site 2 remained the camera port, Sites 4, 5 and 7 were 8‐mm instrument ports, site 6 a 12‐mm insufflation port, and Site 8 a 5‐mm assistant port. The arrangement is the standard one used for robot‐assisted distal gastrectomy at our institution and was not mirrored. (d) The matured ileal conduit stoma in the left lower quadrant. The urinary diversion was performed through a short midline mini‐laparotomy: the gastric team isolated a 20‐cm ileal segment and restored bowel continuity by a functional end‐to‐end anastomosis, and the ureters were joined to the conduit by the Wallace technique over stents.

The gastric team operated first. Pneumoperitoneum was established through an open incision at the umbilicus, and five further ports were placed across the upper abdomen in our standard, non‐mirrored arrangement. Only the table tilt was reversed to 15° head‐up and 12° right‐side up. Distal gastrectomy with D2 lymphadenectomy, cholecystectomy, and Billroth I reconstruction was performed, with a console time of 3 h and 33 min.

The robot was undocked, the lateral tilt was removed, and the patient was placed at 25° head‐down. Four sites were reused, including the camera port at Site 2, and two were added. Bilateral pelvic lymphadenectomy and radical cystoprostatectomy were performed with a console time of 3 h and 5 min; both ureteric margins were negative on frozen sections. Urinary diversion was extracorporeal through a short midline mini‐laparotomy. The stoma was matured in the left lower quadrant, to which the mirrored mesentery brought the terminal ileum (Figure 3d). The total duration of surgery was 10 h 27 min, and the estimated blood loss was 126 mL.

On the second postoperative day, repeated vomiting and hypotension developed; radiography showed marked small bowel dilatation and computed tomography intramural and portal venous gas without arterial occlusion, indicating suspected non‐occlusive mesenteric ischemia. No intraoperative hypotension or vasopressor use was recorded. Nasogastric decompression, fluid resuscitation, intravenous papaverine, and enoxaparin were given; the gas had regressed the next day, and oral intake was resumed on Day 15. The entire bladder mucosa contained no residual carcinoma, and the prostate and all 13 pelvic nodes were negative (ypT0N0). The stomach contained a 38 × 31 mm signet‐ring cell carcinoma invading the muscularis propria, with 13 of 37 nodes involved (ypT2N3aM0, Becker regression Grade 3) [6]. Four adjuvant cycles were administered at reduced dose. Liver metastases appeared 12 months after surgery; CD56 and INSM1 positivity with absent HNF4α, claudin‐18, and TROP2 expression favored a bladder over a gastric origin (Table 1). The patient died 27 months after surgery.

## 3 Discussion

For robotic gastrectomies reported for situs inversus totalis, the ports are usually mirrored [2, 7, 8]. Articulating instruments work from either side, and our gastric team kept its standard arrangement, reversing only the table tilt, which left the ports usable by the urological team.

The pelvis is essentially symmetrical, and although a previous robot‐assisted cystectomy in this anomaly used mirror‐image ports [3], our pelvic dissection required no modification. The practical consequences lay in the bowel: the right ureter was passed behind the sigmoid mesentery, and the terminal ileum and the stoma lay on the left, as in the first reported urinary diversion in this anomaly [9].

Simultaneous robotic resection of two organs in situs inversus totalis has been described once, for the stomach and sigmoid colon [4], and combined robotic procedures across specialties remain few [10]. To our knowledge, the two have not previously been combined in one session. Four of the eight sites, including the camera port, served both procedures, so that neither a second set of incisions nor a second port map was required. The robot was undocked and redocked, but the change‐over required only removal of the lateral tilt and head‐down positioning.

A single case cannot establish that the two procedures can safely be combined; the suspected non‐occlusive mesenteric ischemia after more than 10 h illustrates the risk. Although the entire bladder mucosa was free of tumors, liver metastases attributed to bladder carcinoma appeared a year later, and ypT0 did not preclude hepatic relapse.

Sharing most port sites allowed two major robotic resections to be completed in a single session in situs inversus totalis, but whether this is preferable to staged surgery remains uncertain.

## Ethics Statement

The authors have nothing to report.

## Consent

Written informed consent for publication was obtained from the patient.

## Conflicts of Interest

The authors declare no conflicts of interest.

## Data Availability

The data that support the findings of this study are available from the corresponding author upon reasonable request.
